# Predictors of Medical Mistrust Among Surrogate Decision-Makers of Patients in the ICU at High Risk of Death: A Pilot Study

**DOI:** 10.1016/j.chstcc.2024.100092

**Published:** 2024-08-08

**Authors:** Scott T. Vasher, Jeff Laux, Shannon S. Carson, Blair Wendlandt

**Affiliations:** Division of Pulmonary and Critical Care Medicine, Department of Medicine, University of North Carolina School of Medicine, Chapel Hill, NC; North Carolina Translational and Clinical Science Institute, University of North Carolina, Chapel Hill, NC; Division of Pulmonary and Critical Care Medicine, Department of Medicine, University of North Carolina School of Medicine, Chapel Hill, NC; Division of Pulmonary and Critical Care Medicine, Department of Medicine, University of North Carolina School of Medicine, Chapel Hill, NC

**Keywords:** critical illness, medical mistrust, palliative care, surrogate decision-maker

## Abstract

**BACKGROUND:**

Medical mistrust may worsen communication between ICU surrogate decision-makers and intensivists. The prevalence of and risk factors for medical mistrust among surrogate decision-makers are not known.

**RESEARCH QUESTION:**

What are the potential sociodemographic risk factors for high medical mistrust among surrogate decision-makers of critically ill patients at high risk of death?

**STUDY DESIGN AND METHODS:**

In this pilot cross-sectional study conducted at a single academic medical center between August 2022 and August 2023, adult patients admitted to the medical ICU and their surrogate decision-makers were enrolled. All patients were incapacitated at enrollment with Sequential Organ Failure Assessment scores of ≥ 7 or required mechanical ventilation with vasopressor infusion. Surrogate decision-maker sociodemographic characteristics were age, race, sex, education, relationship to the patient, employment, prior exposure to a loved one transitioning to hospice or comfort-focused care, and religiousness. The primary outcome was surrogate decision-maker medical mistrust, measured using the Medical Mistrust Multiformat Scale. Multiple linear regression was used to determine sociodemographic characteristics associated with higher medical mistrust.

**RESULTS:**

Thirty-one patients and their surrogate decision-makers were enrolled during the study period, surpassing our goal of 30 pairs and indicating recruitment feasibility. Mean ± SD surrogate age was 53.8 ± 14.5 years, 24 surrogates were female, and mean medical mistrust score was 17.1 ± 5.4. Race was associated with medical mistrust, with Black participants showing higher medical mistrust compared with White participants (β =10.21; 95% CI, 3.40-17.02; *P* = .010). Religiousness was associated with lower medical mistrust (β = −2.94; 95% CI, −4.43 to −1.41; *P* = .003). Prior exposure to hospice or comfort-focused care was associated with higher medical mistrust (β = 7.06; 95% CI, 1.21-12.91; *P* = .025).

**INTERPRETATION:**

We found that recruiting ICU surrogates and measuring medical mistrust within 48 h of ICU admission was feasible. Several surrogate sociodemographic characteristics were associated with changes in medical mistrust. These preliminary findings will inform the design of future studies.

Medical mistrust is defined as a sense of unease or suspicion that a health care provider or entity may not act in the patient’s best interest.^1,2^ Mistrust in the US health care system has been rising over the past 50 years.^3^ Since the start of the COVID-19 pandemic, further rapid erosion of trust has occurred,^4,5^ with a Pew survey in 2023 finding that 25% of adults have a great deal of confidence in medical scientists to act in the best interests of the public, down from 43% in 2020.^6,7^

Medical mistrust has consequences for patient care, including worsened communication with providers,^8^ lower quality of life among patients with serious illness,^9^ underuse of health services,^10^ and vaccine hesitancy.^11,12^ For patients belonging to historically underrepresented racial or ethnic groups or those who have experienced homelessness, medical mistrust is a barrier to high-quality goals-of-care conversations.^13-16^ Further, Black patients with serious illness have reported high levels of medical mistrust, exacerbated by experiences of racism during hospitalization.^17^

Goals of care and other serious conversations are common for patients admitted to the ICU and occur frequently between a health care provider and a surrogate decision-maker because of patient incapacitation.^18^ The conceptual model of trust in the ICU describes a balance between surrogate decision-maker trust in ICU providers and mistrust in the health care system.^19,20^ This balance is a construct that originates from surrogate decision-maker lived experiences and preconceptions, some of which are reflected in their sociodemographic characteristics.^21,22^ The model suggests that low trust in ICU providers, high mistrust in the health care system, or both affect how surrogates make decisions and communicate with the ICU team. In turn, changes in surrogate decision-making and communication may impact ICU length of stay, quality of life, quality of the death or dying process, and disparities in care for the patient.^19,23^ Predicting which surrogate decision-makers are at highest risk of medical mistrust is important for addressing surrogate mistrust to improve patient outcomes.

To our knowledge, predictors of medical mistrust have not been determined in surrogate decision makers of patients at high risk of death in the ICU. To demonstrate feasibility of this assessment and to inform larger studies, we conducted a pilot cross-sectional study enrolling surrogate decision-makers and patients to measure surrogate decision-maker medical mistrust and to form preliminary observations about the association between surrogate mistrust and patient outcomes. We hypothesized that ICU surrogate decision-maker medical mistrust can be measured within 48 h of patient ICU admission and that specific surrogate sociodemographic factors are associated with higher levels of surrogate medical mistrust. We conducted an exploratory analysis of recruitment and acceptability to assess full study feasibility.

## Study Design and Methods

### Study Design and Setting

We conducted a cross-sectional study of surrogate decision-makers of patients at high risk of death admitted to the medical ICU at the University of North Carolina Medical Center, an academic quaternary care hospital. A surrogate decision-maker was defined as the person designated either by the patient or applicable state law as being responsible for health care decision-making in the event the patient becomes incapacitated. High risk of death was defined as Sequential Organ Failure Assessment score of ≥ 7 (associated with > 20% ICU mortality) or requiring mechanical ventilation with concurrent administration of vasopressors.^24,25^ Patients at high risk of death were chosen because these patients are more likely to require support from a surrogate decision-maker.^26^ The study protocol was reviewed by the University of North Carolina Biomedical Institutional Review Board (Identifier: 22-1468) and was approved on June 30, 2022. All study procedures were completed in accordance with the institutional review board and the tenets of the Declaration of Helsinki of 1975.

### Study Participants: Inclusion and Exclusion Criteria

Patients and their surrogate decision-makers were enrolled for this study. All patients admitted to the medical ICU were screened briefly using the following inclusion criteria: 18 years of age or older, high risk of death as defined above, lacking decision-making capacity, and admitted to the ICU for < 48 h. Patients receiving moderate or deep sedation or those with a diagnosis of encephalopathy or delirium were considered to lack decision-making capacity. For patients without documentation about decision-making capacity, the determination was made by the primary medical team, rather than the research team.

Patients who passed the brief inclusion screening were screened further for the following exclusion criteria: receiving comfort-focused care, no available surrogate decision-maker, lacking English language proficiency, primary care team attending or fellow discretion, or residing within a department of corrections. Target enrollment for this study was 30 patient-surrogate decision-maker pairs to allow sufficient statistical exploration to assess candidate variables for a fully powered study and to demonstrate feasibility of enrollment.^27^ Power analysis was not conducted because of the pilot nature of this study.

### Enrollment and Data Collection

Patient-surrogate decision-maker pairs meeting inclusion and exclusion criteria were approached for enrollment either at the bedside or by telephone if the surrogate decision-maker was not present at the bedside. Surrogate decision-makers provided written (if approached in person) or verbal (if approached by telephone) informed consent to participate in the survey. Because all patients lacked decision-making capacity at the time of enrollment, surrogate decision-makers were asked to provide consent on behalf of their loved one to participate. The survey instrument was administered in paper format for surrogates approached in person or was distributed electronically for surrogates approached by telephone. Patients who recovered their decision-making capacity before hospital discharge were approached for consent at that time. No contact after discharge was required for this study.

### Feasibility Measures

Recruitment and survey completion metrics were measured to assess feasibility.^28^ Recruitment pace was defined as the number of surrogate decision-makers who consented to participate in the study per 1 month of recruiting. Enrollment success was measured as the number of surrogate decision-makers approached for enrollment compared with the number enrolled successfully. Survey completion was defined as the percentage of surveys that were returned with no incomplete responses.

### Exposures: Surrogate Decision-Maker and Patient Characteristics

Sociodemographic characteristics of surrogates to explore as candidate risk factors for medical mistrust were selected based on prior literature and our conceptual model.^19,29,30^ Demographic characteristics selected were age, sex, race, ethnicity, level of education, employment status, and relationship to the patient. Social characteristics were religiousness and prior exposure to a loved one transitioning to hospice or comfort-focused care. Religiousness was measured on a scale of 0 to 10, with 0 indicating not at all spiritual or religious and 10 indicating very spiritual or religious. Patient characteristics collected were Acute Physiology and Chronic Health Evaluation II score at time of enrollment, COVID-19 infection status, and whether a palliative care consultation was requested during hospitalization.^31^ Sequential Organ Failure Assessment scores were used to screen for potential enrollment because these scores are calculated and displayed for all patients by the electronic medical record, reducing the need to access patient information under a Health Insurance Portability and Accountability Act waiver for screening purposes. After consent was obtained, Acute Physiology and Chronic Health Evaluation II scores were calculated for each enrolled patient to estimate mortality without the need for serial assessments.^24,31^

### Outcome: Medical Mistrust

The primary outcome in this study was surrogate decision-maker medical mistrust. Medical mistrust was measured using the Medical Mistrust Multiformat Scale (MMMS), a six-question validated survey instrument for quantifying medical mistrust on a scale ranging from 6 to 34, with higher levels suggesting increased mistrust. The survey contains questions such as, “Do you feel like medical authorities are trustworthy and honest?,” “How often do you think medical authorities make mistakes?,” and “How much would you agree that medical authorities are more concerned about making money than taking care of people?” No cutoff exists for categorization of medical mistrust scores as low or high. In the original validation study, mean ± SD medical mistrust scores of 17.95 ± 5.62 and 18.40 ± 5.79 were identified in two study populations, the first comprising primarily of Black and White patients with hypertension, and the second comprising a diverse group of patients with diabetes, hypertension, or both.^32^ The MMMS is new, published in 2022, compared with the commonly used Medical Mistrust Index.^10^ The MMMS was selected because it has stronger convergent validity than the Medical Mistrust Index.^32^

### Statistical Analysis

Descriptive statistics were used to report surrogate and patient characteristics and medical mistrust scores. Multiple linear regression was used to determine sociodemographic characteristics that were associated with higher or lower levels of surrogate medical mistrust. All surrogate sociodemographic variables were used in the regression model. It should be noted that in this analysis, the use of race does not reflect our team’s belief that race is a biological distinction that intrinsically changes medical mistrust; rather, it was used in this study to indicate a set of lived experiences and a reflection of long-standing systemic racism that may lead to alterations in medical mistrust in underrepresented and racial and ethnic minority groups.^33^ A *P* value of < .05 was considered statistically significant. Statistical analysis was conducted using STATA version 18 software (StataCorp LLC).

## Results

### Enrollment and Feasibility Results

**Figure 1 –:**
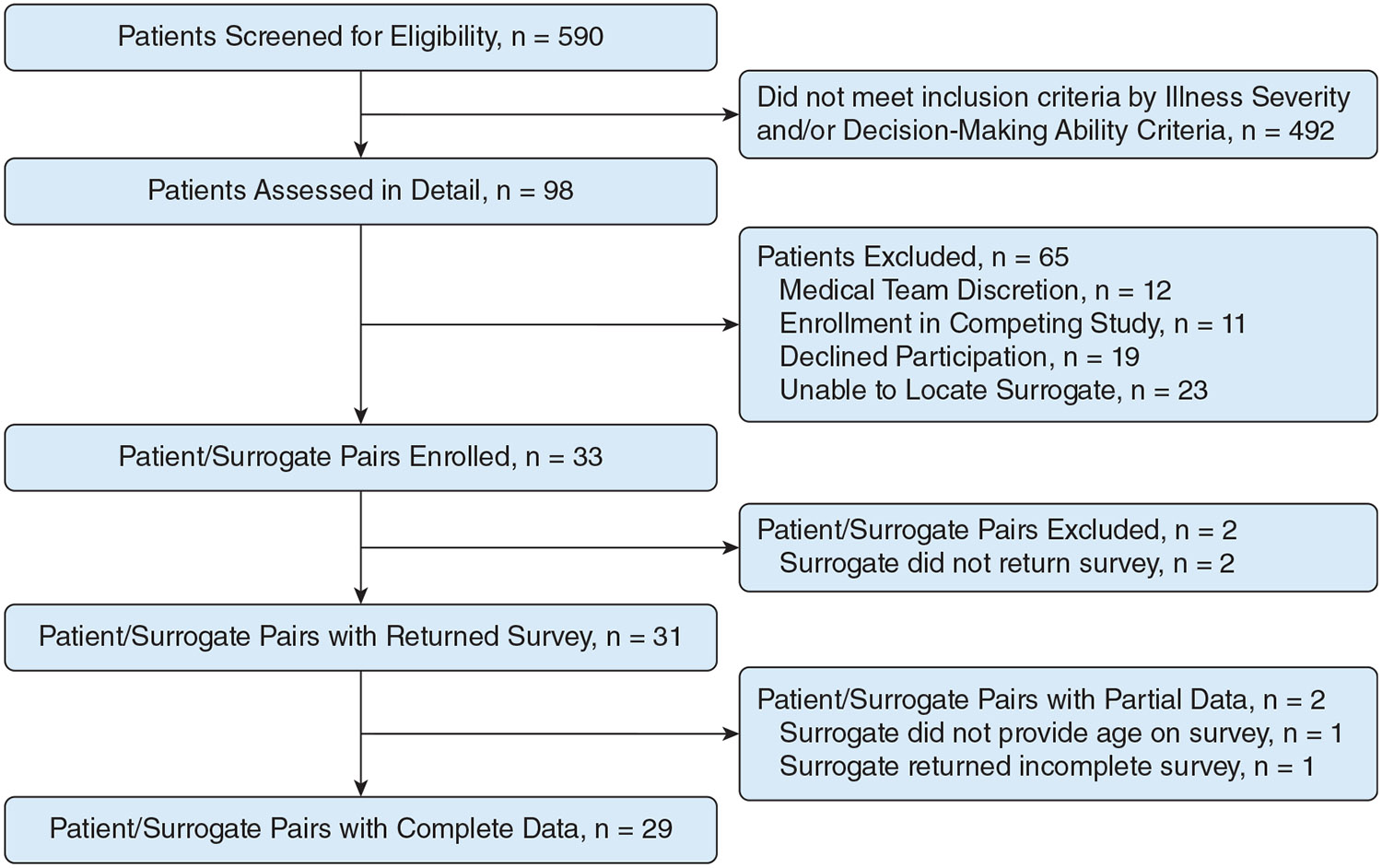
Consolidated Standards of Reporting Trials diagram showing screening and enrollment in the study.

Study participants were enrolled between August 24, 2022, and August 18, 2023. Of the 590 patients screened, 98 patients (17%) met inclusion criteria, of whom 12 patients were excluded by primary team discretion and 11 patients were enrolled in a competing study. Of the remaining 75 patients, 47 patients (63%) had family at the bedside available for approach and 29 patients (39%) agreed to participate. Of the 28 patients without family available at the bedside, successful contact was made by telephone with five surrogate decision-makers, of whom four agreed to participate, but only two completed the survey. Of 52 surrogate decision-makers approached, 33 surrogate decision-makers (63.4%) consented and 31 surrogate decision-makers (59.6%) completed enrollment by returning the survey, 29 (93.5%) of whom completed the survey in its entirety (Fig 1). The pace of enrollment, including all 33 consented surrogate decision-makers, was 2.8 per month. Of the 19 surrogate decision-makers who were approached and declined to participate, the most common reason suggested to the study recruiter for not participating was being too overwhelmed. No surrogate decision-makers made statements suggesting that medical mistrust was a reason for declining to participate.

### Surrogate Decision-Maker and Patient Characteristics

The mean ± SD age of the surrogate decision-makers and patients was 53.8 ± 14.5 years and 57.9 ± 16.7 years, respectively. Twenty-four surrogate decision-makers (77.4%) were female. Fourteen surrogate decision-makers (45.2%) were White, 10 surrogate decision-makers (32.3%) were Black, and two surrogate decision-makers (6.5%) reported being multiracial. Mean ± SD Acute Physiology and Chronic Health Evaluation II score of patients at time of enrollment was 22.1 ± 6.2, and four (12.9%) of them had a COVID-19 infection noted at time of enrollment.^31^ Of the 31 patient-surrogate decision-maker pairs enrolled, 12 patients (38.7%) died with comfort-focused care measures in place, five patients (16.1%) died without comfort-focused care measures in place, and 14 patients (45.2%) improved and left the ICU. The mean ± SD surrogate MMMS score was 17.1 ± 5.4. Surrogate decision-maker characteristics are shown in Table 1, and patient characteristics are shown in Table 2.

### Predictors of Medical Mistrust

In multiple linear regression modeling, we found insufficient evidence to establish an association between surrogate decision-maker age and sex with medical mistrust. Evidence was found of an association between the remaining sociodemographic factors assessed and medical mistrust. Race was associated with surrogate medical mistrust: compared with White surrogate decision-makers, Black surrogate decision-maker race was associated with higher medical mistrust (β = 10.21; 95% CI, 3.40-17.02; *P* = .010). The surrogate decision-maker’s relationship with the patient was also associated with medical mistrust. Compared with being a spouse or partner to the patient, being an adult child of the patient was associated with lower medical mistrust (β = −7.09; 95% CI, −13.04 to −1.15; *P* = .027), as was being a sibling (β = −8.36; 95% CI, −13.30 to −3.42; *P* = .006). Surrogate decision-maker employment status was associated with medical mistrust. Compared with working full-time, working part-time was associated with lower medical mistrust (β = −10.53; 95% CI, −17.61 to −3.45; *P* = .011) and being retired also was associated lower medical mistrust (β = −8.38; 95% CI, −16.15 to −0.61; *P* = .039).

Surrogate decision-maker level of education was associated with medical mistrust, with higher levels of education beyond high school generally associated with lower medical mistrust. Compared with surrogate decision-makers with a high school level of education, those with a bachelor’s degree showed lower medical mistrust (β = −12.22; 95% CI, −21.12 to −3.33; *P* = .015). Self-reported spirituality or religiousness was associated with lower medical mistrust: a one-unit increase in the religiousness scale (range, 0-10) was associated with a decrease in medical mistrust score by 2.94 (95% CI, −4.43 to −1.41; *P* = .003). Surrogate decision-makers who previously had a loved one receive hospice or comfort-focused care were more likely to have higher medical mistrust (β = 7.06; 95% CI, 1.21-12.91; *P* = .025). Linear regression results are shown in Table 3.

## Discussion

In this study, we measured medical mistrust among surrogate decision-makers of critically ill patients at high risk of death, identified surrogate characteristics that were associated with higher and lower levels of medical mistrust, and demonstrated feasibility for a future larger study. Although the pilot, exploratory nature of our analysis means these results should be interpreted with caution, several interesting findings will inform development of a larger study. Consistent with prior studies of medical mistrust,^34-36^ we identified race as a predictor of medical mistrust. This likely represents the effects of long-standing and ongoing structural racism and injustice,^37-40^ particularly among Black Americans. Future interventions should take a race-conscious, critical race theory-informed^41^ approach to mitigate the effect of lived experiences that may be more prevalent in people belonging to underrepresented racial groups and contribute to medical mistrust.

We identified the surrogate decision-maker relationship to the patient as being associated with medical mistrust. Surrogates identifying as a spouse or partner of the patient showed higher medical mistrust compared with adult children or siblings of the patient. Compared with the other sociodemographic characteristics measured in this study, the relationship to the patient characteristic is different because it depends entirely on who the patient is. For example, a surrogate decision-maker could be one patient’s spouse and simultaneously another patient’s child. Would medical mistrust differ in the context of which related patient was admitted to the ICU? Although this finding is novel among patients in the ICU and their surrogates, a comparable construct known as role-based trust has been described in other populations. Role-based trust is the trust that exists based on the roles of two people, such as a patient and medical doctor.^42^ This role-based trust construct could be extended to the relationship between a surrogate decision-maker and patient with respect to medical mistrust and warrants investigation in future studies of medical mistrust.

Little empirical study of the connection between religious belief and medical mistrust has been carried out. A study by Benjamins^43^ examined the relationship between religiousness and trust in health care systems. Although mistrust is not typically considered to be the opposite of trust, many of the survey questions used by Benjamins reflect mistrust constructs comparable with those in the MMMS. Counter to our results, Benjamins^43^ found that strength of affiliation with religion was not associated with (mis)trust in health care systems. One explanation could be changes in medical mistrust, religiousness, or both since Benjamins’s^43^ study, which used data collected in 1998. It is also possible our single-question approach to religiousness was overly simplified, and use of a more granular survey instrument could permit a more nuanced investigation. It is important to note that our results were from surrogates in North Carolina, the 10th most religious state in the country,^44^ and may not be generalizable to other regions.

The most surprising finding is that prior exposure to a loved one receiving hospice or comfort-focused care was associated with higher medical mistrust. Several studies in outpatient and nursing home settings generally have revealed a favorable family view of hospice or palliative approaches to care.^45-48^ A key difference in the ICU is the adjustment period from illness onset to being offered hospice or comfort-focused care compared with those with chronic disease such as dementia and cancer or those residing in a nursing home. In critical illness, the disease process often arises suddenly or without warning compared with chronic illness. One possible explanation for the association between medical mistrust and prior exposure to hospice or comfort-focused care is that regardless of positive or negative prior experience with hospice or comfort-focused care, a surrogate decision-maker may feel mistrustful regarding being offered that option again. Verifying this in future studies is important, as is clarifying which of hospice or comfort-focused care was the driver and if the surrogate played an active decision-making role in this prior instance.

One key goal of this pilot study was to establish feasibility and to optimize methodology for a larger study. Study recruitment took place over 1 year, and 55.8% of surrogate decision-makers who were approached were enrolled successfully and provided complete data for analysis. Many of those who declined to participate expressed feeling too overwhelmed to participate in a study, and none expressed mistrust that affected their participation. Only two participants did not complete the survey entirely, suggesting that most participants found the survey instrument acceptable. The enrollment pace of 2.8 patient-surrogate decision-maker pairs each month was reasonable for a pilot study, but should be optimized in a larger study. Recruitment at multiple geographically diverse sites in future studies could improve generalizability and recruitment pace. Electronic enrollment resulted in four successful telephone enrollments and two complete survey responses. Response rates for paper-and-pencil surveys are known to be higher than Internet-delivered surveys, and this is reflected in our study.^49^ In a meta-analysis of survey methods, the number of contact attempts was demonstrated to improve response rates for web-delivered surveys.^50^ In future studies of medical mistrust, one key enhancement is to extend the enrollment time to 72 h from 48 h to build in time for multiple electronic reminders to optimize enrollment success.

This study has limitations. First, the pilot design of this study means that it was not statistically powered to draw practice-changing conclusions, but our results provide a strong foundation for a fully powered study, which was the intended goal. Second, some of our chosen methods of data collection (for example, the use of a single-item religiousness scale for surrogates or the fact that we did not collect patient race and ethnicity data) limited our ability to explore relevant risk factors for surrogate mistrust fully, and we will obtain more granular assessments of these and other relevant variables in the full study. Third, this was a single-center study limited to participants with English language proficiency (the MMMS^32^ had not been translated and validated in other languages as of the inception of this study), and our sample overall showed low racial and ethnic diversity, limiting generalizability to other locations, payer mixes, and racial and ethnic groups. Future studies would benefit from a multicenter design with an emphasis on inclusive recruitment strategies to maximize participant diversity. Finally, 23 surrogate decision-makers could not be located readily for enrollment, leading to potential selection bias; this (and any) study of medical mistrust is limited by the likely lower participation in research by those with higher levels of medical mistrust.

In this study, we identified multiple sociodemographic factors that may be associated with medical mistrust among surrogate decision-makers of critically ill patients. Identification of surrogate decision-makers at high risk of medical mistrust is a key part of developing interventions to mitigate medical mistrust.

## Figures and Tables

**Table 1 ]:** Demographic Characteristics of ICU Surrogate Decision-Makers

Characteristic	Responders	Surrogates
Age, y	30^a^	53.8 ± 14.5
Female sex	31	24 (77.4)
Race and ethnicity	31	
White		14 (45.2)
Asian or Pacific Islander		1 (3.2)
Hispanic		1 (3.2)
Black		10 (32.3)
Native American or American Indian		2 (6.5)
Multiracial		2 (6.5)
Prefer not to say		1 (3.2)
Relationship to patient	31	
Spouse or partner		14 (45.2)
Parent		4 (12.9)
(Adult) child of patient		7 (22.6)
Sibling		6 (19.4)
Employment status	31	
Employed full-time		18 (58.1)
Employed part-time		5 (16.1)
Retired		5 (16.1)
Unemployed		2 (6.5)
Other		1 (3.2)
Highest education level obtained	31	
High school		5 (16.1)
Trade school		5 (16.1)
Some college		9 (29)
Bachelor’s degree		8 (25.8)
Master’s or doctoral degree		4 (12.9)
Prior exposure to hospice or comfort-focused care	31	
No		15 (48.4)
Yes		14 (45.2)
Prefer not to say		2 (6.5)
Religiousness scale (range, 1-10)	31	10 (8-10)
Medical Mistrust Multiformat Scale score (range, 6-34)	30^b^	17.1 ± 5.4

Data are presented as No. (%), mean ± SD, or median (interquartile range).

a One surrogate decision-maker did not provide their age.

b One surrogate decision-maker did not complete the Medical Mistrust Multiformat Scale questionnaire.

**Table 2 ]:** Patient Demographic and Outcome Characteristics

Characteristic	Patients (N = 31)
Age, y	57.9 ± 16.7
APACHE II score at enrollment	22.1 ± 6.2
Outcome	
Condition improved – left the hospital	14 (45.2)
Passed away with comfort measures in place^a^	12 (38.7)
Passed away without comfort measures in place	5 (16.1)
Hospital length of stay, d	13.4 (2.6-42.1)
Option for comfort-focused care offered	
Yes	16 (51.6)
No	15 (48.4)
Palliative care service consulted	
Yes	6 (19.4)
No	25 (80.6)
COVID-19 present during hospitalization	
Yes	4 (12.9)
No	27 (87.1)

Data are presented as No. (%), mean ± SD, or median (interquartile range). APACHE = Acute Physiology and Chronic Health Evaluation.

a Includes patients discharged with hospice-focused care measures in place.

**Table 3 ]:** Multiple Linear Regression Model of Surrogate Sociodemographic Factors Predicting Medical Mistrust

Surrogate Characteristic	Full Model (n = 29)	
	β Coefficient (95% CI)	*P* Value
Age	−0.07 (−0.34 to 0.21)	.574
Sex		
Male	Reference	NA
Female	0.81 (−8.19 to 9.81)	.833
Race and ethnicity		
White	Reference	NA
Asian or Pacific Islander	−17.87 (−40.36 to 4.63)	.100
Hispanic or Latino	27.32 (15.73 to 38.91)	.001
Black	10.21 (3.40 to 17.02)	.010
Native American or American Indian	−2.05 (−10.24 to 6.14)	.563
Multiracial	−0.52 (−12.77 to 11.74)	.921
Prefer not to say	−24.93 (−47.09 to −2.76)	.033
Relationship to patient		
Spouse or partner	Reference	NA
Parent	−0.50 (−7.85 to 6.85)	.874
(Adult) child	−7.09 (−13.04 to −1.15)	.027
Sibling	−8.36 (−13.30 to −3.42)	.006
Employment status		
Employed full-time	Reference	NA
Employed part-time	−10.53 (−17.61 to −3.45)	.011
Retired	−8.38 (−16.15 to −0.61)	.039
Unemployed	9.38 (−1.96 to 20.72)	.089
Other	11.63 (−2.19 to 25.45)	.085
Highest education level obtained		
High school	Reference	NA
Trade school	−2.91 (−11.66 to 5.84)	.447
Some college	−5.07 (−12.20 to 2.07)	.133
Bachelor’s degree	−12.22 (−21.12 to −3.33)	.015
Master’s or doctoral degree	−0.44 (−11.90 to 11.02)	.928
Prior exposure to hospice or comfort-focused care		
No	Reference	NA
Yes	7.06 (1.21 to 12.91)	.025
Prefer not to say	24.42 (6.85 to 41.99)	.014
Religiousness	−2.94 (−4.43 to −1.47)	.003

Positive coefficients are associated with higher medical mistrust, and negative coefficients are associated with lower medical mistrust. Surrogates with complete data available for analysis are included. Two surrogates returned incomplete surveys, representing 6% of total data omitted from the regression model. NA = not applicable.

## Take-Home Points

### Study Question:

Can plausible risk factors for medical mistrust be identified in surrogate decision-makers for high-risk patients?

### Results:

We found that ICU surrogate decision-maker medical mistrust can be measured using the Medical Mistrust Multiformat Scale, and multiple sociodemographic factors are associated with higher medical mistrust, including race and having a prior loved one who received hospice or comfort-focused care.

### Interpretation:

This pilot investigation into ICU surrogate decision-maker medical mistrust demonstrated that medical mistrust can be measured and sociodemographic characteristics can be identified that are associated with higher or lower medical mistrust.

## ABBREVIATIONS:

MMMS: Medical Mistrust Multiformat Scale
